# Insights for public education provided by French media on ideas about prostate cancer – A media analysis study

**DOI:** 10.15171/hpp.2018.12

**Published:** 2018-04-18

**Authors:** Margareth S. Zanchetta, Marguerite Cognet, Mary Rachel Lam-Kin-Teng, Marie Elisabeth Dumitriu, Carlos Haag, Bernard Kadio, François Desgrandchamps, Lise Rénaud

**Affiliations:** ^1^Daphne Cockwell School of Nursing, Ryerson University, Toronto, Canada; ^2^Unité de Formation et Recherche Sciences Sociales, Université Denis Diderot, Paris, France; ^3^Interdisciplinary School of Health Sciences- University of Ottawa, Ottawa, Canada; ^4^Unité de Formation et Recherche Médicine, Université Denis Diderot, Paris, France; ^5^Faculté de communication, Université du Québec à Montréal, Montréal, Canada

**Keywords:** Health education, Media content analysis, Health messages, Prostate cancer

## Abstract

**Background:** This study explored the French media’s presentation of ideas and medical information about prostate cancer (PC) that may influence men’s understanding, attitudes and behavior.

**Methods:** A qualitative media content analysis centered on PC information delivered by French professional media. The selected data were produced in the aftermath of the High Health Authority's decision in 2008 not to recommend systematic screening by prostate specific antigen(PSA) for men over 50. Source was the Media Archives of the French National Library. Content was analyzed from 15 television programs, 14 radio programs, and 55 articles from 35 popular French newspapers (online and printed, weekly and monthly) and 20 magazines. Audio content was narrated into textual form and submitted to manual coding along with the print content.

**Results:** Television and radio content focused on the nature of PC, screening and treatment,and conveyed a gender-centric position linked to male sexuality and virility. Newspapers and magazines targeted the testing controversy, the lack of consensus among professionals, and scientific advances in screening and treatment.

**Conclusion:** Media participation in the European testing debate is valuable for allowing patients to hear all opinions on PC risk factors. Debate on testing policy contributes to confusion and uncertainty regarding appropriate action.

## Introduction

Globally, media contribution and influence (e.g. newspapers, magazines, newsletters, television, radio, Internet) has the capacity to impact men’s and women’s cancer literacy. Global news increasingly influences the way men and women, at individual and collective levels, understand cancer.^1^ The way the population perceives cancer affects individual preventative attitudes and informs approaches for the design of official cancer control policies.^2^

In the last decades, researchers studied cancer representation in the conventional media, seeking to understand types of news coverage and the public’s knowledge and perceptions about cancer. In an experimental study^3^ on 6 chosen German TV clips (broadcasted over a period of two years), messages with positive and negative connotations were used to describe cancer diagnoses and therapies. In consumers’ information-related evaluations, there was no evidence found of intention to change attitudes and behaviors in response to journalists’ emphasis on news. However, evidence supported assumptions that gain-based information was more effective than loss-based messages. Some authors suggested that media studies should explore the appropriation of the message as a long-term process rather than its contents,^4^ while other authors stated that media power can place cancer prevention and diagnosis on the public agenda, thus impacting public health.^3^

Knowledge about prostate cancer (PC) for men of all ages intertwines with issues of learning preferences and underlying masculinity values. Men from ethno-cultural groups prefer varied types of media; PC screening indicated that radio reached African American men (25%) while newspapers reached mostly the Caucasian ones (34%).^5^ Clarke’s^6^ media analysis study of 36 American and Canadian circulating magazines (published in 1974-1995) revealed the hidden manifestation of the intersection of PC and masculinity.

When men search for PC-related information, the various screening and available treatment modalities may bring disagreement to the patient-health professionals’ joint course of action regarding choice from these various modalities. High complexity medical information exists about methods of PC screening (e.g. digital rectal examination and prostate specific antigen (PSA) blood test,^7^ as well as, protein-based, DNA-based and RNA-based markers)^8^ and treatments (e.g. active surveillance, surgical removal of tumour, conventional and new radiation therapy (e.g. intensity-modulated radiation therapy, stereotactic body radiation therapy, and hadron therapy)^9^ and androgen deprivation therapy).^8,10^ This search for PC-related knowledge through informal sources can promote patients’ autonomy to independently research additional information and actively participate in their care and decision-making.^11,12^ General literacy and health literacy levels may also influence men’s choices of informal sources of information (e.g. media type) because both science-related media and men’s scientific interests are factors which cause transforming attitudes towards the regular use of scientific media.^3^

Men’s understanding of PC information seems to be equally influenced by both media and by friends or family members regarding potential risks from PC screening^13^ despite the known exposure to the screening debate provoked by nonmedical sources of information (e.g. newspapers, magazines, newsletters, television, radio, Internet, family, friends, and coworkers).^14^ In the last decade, authors (mostly North American) have intensified their warnings about the distortions of cancer indicators presented by the media.^1^ For instance, Jensen et al^1^ analyzed 5327 cancer stories in the top 50 US newspapers. A consistent under reporting of male reproductive cancer, lymphatic Hodgkin’s, and thyroid cancers was identified, while breast, leukemia, pancreatic, and bone-muscle cancers have been over-reported. News promoted treatment of these cancers more than prevention strategies, lifestyle choices, and potential risk factors.

Conventional media, however, may be influential sources of PC-related information that can be useful and actionable. The conventional media also reinforces ideals of male sexuality and masculinity when discussing PC and its treatment. North American magazine articles highlighted sexuality and masculinity,^15^ reinforcing the image of PC treatment and cancer as a ‘threat to sexual function’ due to the side effects of impotence and incontinence. While perpetuating a perceived loss of masculinity when portraying the success of PC treatments, Canadian newspapers’ negative descriptions of men living with PC as weak and frail ironically co-exist with positive body image terms depicting men who underwent PC treatment as those with a “clear bill of health” (p. 160).^16^ Again, in a more recent study on a qualitative analysis of social determinants of men’s health in popular Canadian newspapers, PC remained reported as a popular men’s health issue.^17^

In the United States, exposure to media sources (television, radio, newspaper, magazine and pamphlet) on cancer screening and prevention behaviours (including for PC and PSA screening) was documented as indistinguishable from an influential conversation with friends since all sources may have a synergetic action.^18^ Men (mostly highly educated) reporting positive experiences with information-seeking in the media about uncertainty on treatment choices for slowing-growing PC, PSA test inaccuracy, and treatment side effects; they demonstrated a negative outcome on fatalistic cancer beliefs being a strong predictor of levels of trust on physicians, as well as men’s roles as informed decision-makers.^19^

In a 2-year period on PSA screening, amidst the conflicting recommendations available in US newspapers and TV news, it was documented that such types of conflicting information undermined men’s intentions for seeking information about PSA testing among those who did not get their tests in a previous year. The use of nonmedical sources was only reported by 11% of men, and they ranked seeking information in the media as the third (for newspapers, magazines and newsletters) and fourth (for television and radio) sources they employed.^14^

Today, Anglo-Saxon countries continue to target conventional mass print media (newspapers and magazines), as well as social media, including tutorial videos (among others). The potential for raising awareness and enhancing understanding through men’s discussions using Twitter has proved unremarkable, representing 0.7% of 12 666 tweets in North America and United Kingdom.^20^A more recent trend in media studies on PC involves analyzing Youtube videos (n = 100), of which the majority had been uploaded by consumers (45%) with the purposes of providing information (78%), discussing PC treatment (51%), and presenting PSA testing and routine screening (26%).^21^

Within the European context of men’s health promotion, PC remains a growing problem due to the challenges of obtaining an effective diagnosis and control based on a complete understanding of its causes.^22^ In France, PC is also a significant health concern, with 53 913 new cases and 8893 deaths estimated to have occurred in 2011.^23^ PC early detection information should target men’s sustainable proactive health behaviours since recent evidence about cancer awareness campaigns revealed that men do not sustain long-term interest and discussion in social networks.^24^

In 2015, an international debate about the professional media’s contribution to public awareness of cancer was instigated by the spectacular polemics about the haphazard nature of developing cancer. These revealed the incongruence of information rapidly broadcasted by professional media, medical associations, and social media, including the World Health Organization.^25^ These polemics also uncovered the French context regarding cancer-related information provided to the public, where collective silence from health professionals and other public social actors on social networks and Internet occurs, while all of whom could promptly correct any misinformation and educate the population about essential preventative rules. Moreover, the absence of a common position from the scientific community and the lack of a coordinated response by health authorities were also at play.^26^

As one component of a larger ethnographic study about a transcultural representation of PC among men within a French cultural context, this paper reports a distinctive set of results of a media analysis study. This media study aimed to analyze published and broadcasted media messages aimed at public PC education that can be assumed to reach a global French-speaking audience. The research question “*What ideas about PC are disseminated by written and audio-visual media produced in Paris?”* prompted an exploration of media messages that may influence men’s representation of PC based on the medical information presented, and consequently, men’s attitudes and behaviours.

The results may be informative to professionals interested in tailoring cancer control programs to multicultural male populations, and may provide useful information for the design of systematic, evidence-based programs for prevention, early detection, diagnosis, treatment and palliation by optimizing the use of available resources.^27^

## Materials and Methods

The data for this media content analysis study were gathered in the French National Library (the François Mitterrand site in Paris). The first author consulted the Media Archives and retrieved media content produced after the controversial 2008 decision of the High Health Authority (HHA) (city of Paris) to not recommend systemic PC screening by PSA for men over 50. She retrieved and watched 15 television programs and listened to 14 radio programs about PC that were broadcasted between January 2011 and May 2013. In her exploratory work, the first author accessed all programs about PC broadcasted in this period without applying any criteria of eligibility. She also reviewed 55 articles from popular French (online and printed) newspapers (n = 35) and magazines (n = 20) (2008-2013). Newspaper e-files were downloaded. Content from television and radio productions was transcribed into a fieldwork log as narrative texts, assembled according to a preliminary retrieval table as recommended by Leray^28^ and Renaud^29^ for the analysis of media content. These texts were analyzed by the first, second and third authors.

The qualitative method of analysis of the retrieved texts focused on theme, type of content and media, key message, idea trends (whether the tone of language was informative, alarmist, sensationalist, educative, critical or political) and supporting visual material (pictures, diagrams, images). Both types of material were read and manually coded by first, fourth and fifth authors. These authors conducted an analysis focused on the statements made by journalists, general physicians (GPs), urologists, psychologists, patients, call-in listeners, as well as health authorities. As for the larger study, the analysis was framed by the Core of Social Representation Theory.^30,31^ A social representation is a way of knowing and a way of social thinking, both of which include common sense. It is related to communication, understanding of lived experiences, and mastering one’s cultural, social, ideological and material environment.^32^ The core of a representation is stable regardless of new experiences or knowledge, but it can generate or change the meaning and the value of a given social representation. The core representation organizes the links between elements of the social representation. These elements are most likely to be modified by one’s interpretation of the outcome of new experiences and learning within the social world.

Key messages about PC were extracted (see Table 1) and advisory statements regarding PC prevention and control were recorded (see Table 2). Final interpretation of the analyzed findings was reviewed and confirmed by the other co-authors, who are familiar with the French media.

**Table 1:** Key messages about prostate cancer prevention/control as disseminated by the French media (period 2008-2013)

**Source: Television**	**Source: Radio**	**Source: Newspaper & Magazine**
Digital rectum examination should be performed with alternative screening methods. Scientific innovations support new methods of screening, treatment and rehabilitation. New scientific evidence reveals the occupational risks faced by rural plantation workers due to the use of agro-toxics. The French legal system acknowledges PC-related hazards due to toxin exposure affecting rural workers. Despite emotional impact of the diagnosis including shock and experience of lack of understanding, patients should not despair.	PSA screening is considered ‘bad’ because it can increase mortality rate. PC may be triggered by biopsy. PSA is bad for diagnosis but excellent for follow-up. Cultural factors support the idea of cancer treatment but undermine the idea of active surveillance and follow-up. Individual approach and active surveillance have collective and economic value. Potential new method of PC identification using dogs to sniff urine odor to identify the presence of sarcosine. Importance of consulting with physician for one’s PSA follow-up. Ministry of Health updates on reimbursement of costs for the drug Jetvana for advanced stages of PC. Many African listeners denounced the lack of access and treatment options in their regions, even inquiring about the possibility of consulting a French physician, and thus representing a potential for migrant PC patients.	Risk factors include genetics, medications/supplements, chemicals and possibly certain infectious agents. Sexual abstinence has no effect on PC risk. PC protective factors include a healthy diet and circumcision. Scientific studies are advancing on PC screening and its evolution. Since PC evolves slowly, active monitoring is recommended. Treatment is required in case of fast evolution. Active monitoring should not scare patients if well explained.

**Table 2:** Advisory statements about PC prevention and control

**Source: Television**	**Source: Radio**	**Source: Newspaper**	**Source: Magazine**
Many forms of treatment are available, the problem is how to choose the best one. The national association of PC patients can help. Dialogue with the patient is necessary in the choice of a treatment to treat a specific cancer. Cancer aggressiveness and consequences, and the patient’s concerns about his life and his sexual expectations are considered. Abdominal obesity and prostate enlargement after the age of 50 are risk factors. Ejaculation is believed to protect against PC, but multiple sexual partners exposes one to risk of urological infections and PC.	Guilt about PC is in men’s minds. The disease is fatal. Men should be discreet among their social acquaintances since people tend to inquire about wrongdoing as the PC cause. Even in couples, guilt may be linked to infidelity, but they should overcome such thoughts to jointly deal with PC. Today there is a real revolution in handling PC because once one gets the diagnosis, treatment is not necessarily initiated. Ejaculation problems may not be solved, and erection dysfunction depends on the surgical technique. Recovery may take from 1 month to 3 years since nerve preservation will determine resumption of erections.	The usual French diet provides satisfactory nutrition…the correct attitude is to improve any poor diet habits. A high PSA simply means that something is happening in the prostate - cancer or something else. In some men, this may progress towards a disease and for others, it will never evolve… Risk of incontinence is better managed today, but problems of erection and ejaculation remain…. Currently, early family cancer antecedents are stronger factors for assessing risk than blood genetic markers.	Why resume the debate about the need – or absurdity – of proposing [PSA test] screening for all men who are older than 50 years? Treatments, even if performed by skilled hands, frequently lead to incontinence and impotence. One should not discourage physicians from identifying aggressive cancer cases but restrain the implementation of *non-useful and dangerous* treatments. (italics added) A hyper caloric diet is particularly deleterious…a change in food regimen can slow down the disease’s evolution. Poor scientific evidence linking PC to the absence of sexual intercourse.

## Results

The PSA screening controversy, education about PC prevention, progression, treatment and rehabilitation, as well as recent general scientific findings were the major subjects introduced by French media. Since men’s overall reaction to PC is multidimensional, it was notable to find the absence of any reference to the relevance of men’s religion, masculinity (traditional, metrosexual, etc.), self-identified gender (heterosexual, homosexual, bisexual, transgender, etc.), or men who have no female partner who could serve as a health promoter.

The health television (average 60 minutes) and radio (average 20 minutes) programs had been broadcasted frequently during morning and early evening times, and national and international French-speaking audiences were able to interact with the guest professionals immediately after the broadcasts through the television/radio/internet sites. The newspaper and magazine articles were short and written in accessible language. In this section, the first author’s (the researcher) narrative texts about television and radio broadcasts are presented as verbatim to illustrate the contents of messages on these channels of communication.

### Television

The television programs were informative (n = 14) and educational (n = 10). They provided in-depth explanations about PC evolution, treatment methods, and preventative measures. Interestingly, women were frequently targeted as the primary audience:

Holding 2 small tomatoes, the journalist inquires women what is their role in PC of their husband.^33^ [researcher’s note]

These programs had a strong scientific component and presented international and national advances in PC-related research and treatment. Visual 3D animations, pictorial diagrams, clips, interviews, and statistical information were used to transmit a medical representation of PC. The educational approach guided the public to understand PC in its various trajectories, with the visual aids also acting as a platform for discussion among the health journalists and professionals.

The journalist presents the issue of the controversy in the medical field…that PC has a good prognosis. He asks the epidemiologist if one could improve the survival rate with the screening.^34^ [researcher’s note].

The scientific controversy regarding the clinical usefulness of PSA testing was one of the most prominent topics, but other research news was also widely commented on. The lack of international, scientific consensus about PSA screening and PC treatment was presented as a political schism among GPs, urologists and public health authorities. Although it was not the focus of interest for this study, the Internet seemed to be a space where intense advocacy was expressed by these professionals. In contrast, television messages were clear and straightforward regarding the polemics of individual use of PSA tests versus systemic PSA screening.

The journalist explains the recommendation’s [HHA] reason [for not having a systemic PSA screening] including the unnecessary surgeries with serious urinary and sexual consequences.^35^ [researcher’s note]

Both food and sexuality issues were extensively debated as preventative and rehabilitation subjects; the latter were intertwined with the debate of over-treatment and unnecessary interventions, reinforcing the discrepancy between GPs’ and urologists’ messages. The programs delivered three clear messages from GPs: (*i*) early detection of PC can lead to over-treatment and unnecessary radical prostatectomy, radiotherapy, or hormone therapy; (*ii*) there is a high probability that patients will die from diseases or conditions other than PC; and (*iii*) some male GPs are uncomfortable about proposing or performing digital rectal prostate examinations.

Responses to practical questions addressed how treatment may affect sexual life due to the side effects of surgery, with PC survivors speaking about their personal experiences. Incorporating former patients’ testimonies and lived experiences corroborated or challenged professional viewpoints, and extended the discussion to include the critical role of communication among healthcare teams and patients throughout the PC trajectory.

The journalist states “When one says: ‘you have prostate cancer; it’s not serious’, something is disturbing in this sentence”. He suggests that together we can work on the words to talk about that…like the colon cancer…a polyp.^36^ [researcher’s note].

Many of the programs discussed “unnecessary [medical] procedures” as a risk factor, but with a narrow focus and personal testimonies that did not venture beyond sexual issues. While the programs addressed how to solve such problems, they did not extend the discussion beyond them. The participation of PC survivors added a tone of realism mixed with optimism; their personal testimonies covered their inattention to health prior to a diagnosis of PC and their experiences throughout medical treatment, with brief comments on their self-management solutions for treatment consequences.

The patient says that neither the disease nor the treatment are handicaps in his life. He says that still practice sports.^37^ [researcher’s note].

### Radio

Radio programs were more accessible to audiences and provided interactive opportunities to ask questions or discuss points directly with invited professionals and journalists. The programs targeted PC itself and not the polemics of PSA testing. Their discourse was generally informative (n = 8) and educational (n = 6), with direct questions posed by listeners and short answers from professionals.

This question-and-answer format did not allow for a complete discussion on any one topic; rather, several topics were discussed in less than 30 seconds each, like information bullets as done by two Paris listeners:

…whether the prostate infection may cause prostate cancer? The urologist explains that there is a relation between repeated prostatitis and prostate cancer.^38^ [researcher’s note]

…so, drink a lot of milk causes prostate cancer and eat a lot of tomato is risky to provoke prostate problems, which health practice to avoid it?^39^ [researcher’s note]

Open to listeners everywhere, the radio was the only medium where African men’s issues regarding PC were properly addressed, with requests for information coming from many African countries. The focus of discussion was practical: “How PC affects my life; “What can I do?” and so on. Few of the radio programs tackled PSA screening debate, and when done, it was with balanced points-of-view, as in the following researcher’s notes.

A Brazzaville listener asks if there is a link between PC and intense sexual life. The urologist responds that actually 3 to 4 years ago we believed that not.^40^

A patient from Mali asks about the difference between adenoma, PC and hereditary factors.^40^

Frequently more GPs than urologists were guests (including very few sexologists) and responded to the listeners’ questions as asked by a 70 years-old Paris listener:

…Several high PSA results and no positive biopsy. He knows that today they use only MRI [magnetic resonance imagery] to identify PC. He has a pacemaker and to him, MRI is strictly prohibited. He asks if there is other accurate way to detect PC.^41^ [researcher’s note].

### Newspapers

Newspaper journalists (which included 5 who were identified as physicians and researchers) wrote about an array of PC-related subjects. The PSA controversy was widely explored, as well as scientific advances in screening and treatment. Most of the news items were published in 2012 (n = 11), the year that the urologists intensively challenged the HHA report (supported by the French Association of Urology [FAU], the proponent of national screening day since 2005). Consequently, the topic of PSA testing was the feature of most new items (n = 9), alongside frequent references to the risks associated with biopsies and over-diagnosis and overtreatment. Readers were told that certain knowledge gaps justified the HHA decision — the unknown interactions among genetics and environmental risks, and the difficulty of identifying at-risk groups and highly aggressive forms of PC, or men with risk factors for aggressive or rapid evolution forms of PC.

All positions regarding the PSA controversy involving the HHA and urologists’ practice were noted, potentially confusing readers since it seemed to counter messages from physicians and patients. To counterbalance this, journalists brought men into the PSA debate recommended that men discuss PC screening with their GPs.

….their doctrine can be summarized: ‘there is no room to screen prostate cancer because it is inoffensive and its treatment is unjustified’… in reality the over-diagnosis must not be avoided, it’s the overtreatment that must be.^42^

Despite overall recommendations for active surveillance, men’s adherence to it with requests for PSA testing, rectal digital examination and biopsy (if necessary) was reported as a major issue by urologists.

While waiting for a more efficient PSA test, the rectum examination remains the baseline exam.^43^

Newspapers stated the high incidence of prostatectomy may be explained by patients’ reluctance to ask for any type of intervention. The seemingly contradictory medical advice and men’s (arguably) misinformed decision-making may explain this as well.

Two thirds of men to whom we propose this option do accept it but one third after several months “breakdown” and prefer to consider a more radical solution (usually a surgery) despite all its potential downsides.^43^

…after two years the patient does prefer a treatment. Hard to resist to the uncertainty of living with a cancer.^43^

Although they represent a significant proportion of the general French population, men of African descent (known to be a group at risk)^43^ were referred to only once by the HHA President when he recommended a review of new scientific evidence regarding studies to be conducted with men from the Antilles region, whose PC incidence is higher than that for men in the metropolitan regions of France.

[in the African sub-population]…Screening would aim to demonstrate a mortality decrease in a sub-group presenting risk factors.^43^

Some medical experts made polemical statements, for instance, claiming that French physicians were skilled in the art of PC treatment but not in screening for aggressive PC.^44^ They also stated that preventative medicine practitioners should define new rules and screening approaches for better assessments about proposed treatments.

Using plain language, newspapers gave a detailed description of clinical trials and epidemiological studies, as well as the medical process from screening to rehabilitation. Many of the news articles (n = 13) referenced American and European scientific studies to support scientists’ claims about the limited appropriateness and usefulness of PC screening through PSA testing, and clarified the meaning and benefits of active surveillance as well as preventative approaches such as improving nutritional habits. Newspapers warned about an increased risk to PC from the overuse of anti-oxidants and vitamins,^45^ and the ongoing debate about prohibiting chemicals linked to hormone sensitive cancers like PC.

Newspaper articles also clarified the clinical relevance of testing for PC, and ways and challenges to identify PC aggressiveness (n = 3), based on the results of an experimental study that used blood and urinary markers (n = 4) and magnetic resonance imagery (MRI) (n = 4) to replace the use of biopsy and treat early stages of PC, as well as identify genetic variations to target sub-populations at high risk (n = 1) of fatal evolution (n = 1). The PCA 3 screening urinary test was introduced (n = 1) as a complementary test to PSA. The extra cost to patients of this test was noted, and the use of the digital blood drop test was suggested as an alternative. The emphasis was on using an easy test to guide urologists regarding the need for a targeted biopsy. Journalists also wrote about limited access to diagnostic procedures (MRI equipment) that might jeopardize the implementation of new therapies, such as phototherapy, being adopted by other European and American healthcare organizations.^46^ Phototherapy, a novel non-surgical technique, was touted as a potential complementary therapy.^47^ Along with other focal therapies (cryotherapy, and ultrasound) (n = 3), newspaper articles described an initiative that used robots in surgery (n = 1), and explained the logic underlying physicians’ decisions to use focal therapies. One enthusiastic news item outlined how robotic surgery optimizes a surgeon’s precision and reduces the risk of urinary incontinence and erectile dysfunction.

A hopeful tone was used to discuss new drugs [Experimental drugs’ names are not listed in this manuscript] expected to replace surgical intervention (n = 1), the availability of medical treatment for advanced stages of PC (n = 1), and a new surgical technique to correct urinary incontinence (n = 1). The anticipation of positive outcomes was subtly suggested by references to American books that criticize unnecessary prostatectomies, as well as two items about the fund-raising campaign Movember, a social movement in Anglo-Saxon countries (also started in France in 2012) to raise awareness and funding for PC research.^48^

### Magazines

Of the twenty magazine articles on PC, one was published in 2009, 4 in 2011, and 11 in 2012. The PSA controversy was presented as a lack of consensus among epidemiologists, urologists, HHA and GPs. Journalists criticized FAU’s recommendations and supported HHA’s recommendation, and referred to American and European studies underway that may resolve the disagreement.^49^ Magazine articles also covered the HHA president’s conclusions about the lack of scientific evidence to recommend systemic PSA screening.^50^

…early detection by PSA seems indispensable to reduce PC mortality: by an intelligent way of its practice and establish a code of best practices to avoid unjustified prostatectomies.^51^

Nevertheless, some GPs and many urologists continue to prescribe the PSA test for their asymptomatic patients over 50 years of age.^52^ To support the argument that PC is not necessarily responsible for patients’ death, data was presented showing that only 3% of diagnosed PC cases result in death.^53^ This author portrayed prostate biopsy as an aggressive, unnecessary diagnostic procedure causing disastrous secondary effects such as urinary, digestive and sexual problems.^53^ However, urologists stated: “…according to [epidemiological] data there are much more patients who do not need treatment…there is much more patients dying with their prostate cancer than of their cancer [p. 465].^54^

The results of various American, British and French scientific studies were discussed in the magazine articles. These covered a range of relevant scientific evidences: the link between PC and xenotropic murine leukemia virus-related (XMRV) virus^55^;comparisons of prostatectomy *vs* active surveillance and metastasis^51^; circumcision and PC prevention^56^; use of E vitamin and increased PC risk^57^; the high cost of a monthly testosterone production blocker^58^;localized treatment by ultra-sound^59^; the contra-indication of food supplements for men living with PC^60^; evidence the high serum concentration of Chlordecon in Guadalupe PC patients^61^; and advances on addressing radiotherapy side effects by a test (Apoptise Lymphocytaire) that was developed and introduced by the France National Cancer Institute for the early detection of possible lymphocytes destruction due to radiotherapy. Readers were told that this test was used in some hospitals in combination with nuclear accelerators to identify and treat cancer cells without damaging normal cells.^62^

Magazines also addressed social advocacy in articles on the Movember campaign using the moustache as an icon to encourage open public discussion about PC and to raise funds for research; details were provided to guide men to participate.^48^ A summary display of the media communication on PC by source is shown in Tables 1 and 2, with corresponding messages and key statements that outline the findings.

## Discussion

Media participation in the European debate on PSA is valuable in that it allows patients to hear a full range of opinions.^63^ However, the professional debate about PSA policy nourishes a context of lack of consensus, unclear knowledge, and uncertainty regarding the best course of action; moreover, it may jeopardize media effectiveness to inform the public about PC prevention and control. From the European patients’ perspective, the lack of clarity about PC risk factors is a major issue.^64^ The general uncertainty provoked by the lack of medical consensus about PC screening may undermine men’s self-confidence to deal with the complexity and multiplicity of scientific PC-related information.^65^ This is particularly true for decoding research news, which may reinforce men’s perceptions that scientific information is beyond their understanding and it is overwhelming to try. This is an especially critical issue for those with low health literacy.^66^ In the United States, authors wondered if the influence of media reporting the controversy of PSA screening might be a possible cause for at-risk men’s decreased adherence to this practice, mainly for those men who were also less literate.^67^

The television and radio programs focused on the nature of PC, screening and treatment, but from an intensively focused, gender-centered position that linked PC to sexuality and virility that distorts a constructive approach to prevention and control.^68^ In the Western nations of Europe, including France, traditional media (television, radio, magazines and newspapers) had little effect on increasing the knowledge of screening benefits, including PC screening. However, the use of aforementioned media for health-information seeking in France was above the European average by the following order: TV, magazines, newspapers and radio.^69^

In contrast, Japanese newspaper articles in a year (n = 5314) most frequently presented treatment information and social issues, while prevention and screening were rarely mentioned. PC-related articles for adults were less reported (2.7%) despite being ranked as the fifth incidental type of cancer in Japan.^70^ Since newspapers there are a popular source of information for the general public, the authors recommended that evidence-based cancer information should be provided to prevent misunderstandings.

On the other hand, these programs’ ability to reach international French-speaking audiences in a global health context and to clarify misconceptions by use of virtual/indirect encounters between men and professional experts may counteract the confusing effects of apparently contradictory medical advice. Health care professionals should facilitate men’s access to reliable information about susceptibility and severity, specific to their health matters.^71^

Given the current level of global connectivity, media platforms have the ability to reach large populations. However, with the increasing popularity of mobile internet and social media, information dissemination is continuously transforming. Mobility of health information is now a key factor^72^ in reaching diverse groups of men within the general population, so the potential of radio programs and mobile versions of printed media should be utilized to promote wider coverage of PC-related news. For instance, the debate about cancer over-diagnosis and over-treatment as discussed in France^54,73,74^ is a global phenomenon.^63^ French radio and television (TV5 Monde)^75^ reaches a global audience, making it a potentially significant force in educating lay populations, especially those immersed in an oral culture. The need to address the global audience including males of African descent is pressing, especially given the paucity of scientific evidences of that in host societies.

## Limitations and Strengths

Throughout the fieldwork of this ethnographic study, a PC survivor who could mention having been exposed to any form of the audio-visual and printed media could not be found, which thus undermined the verification of the findings’ interpretation. Therefore, men’s representation of PC, based on the medical information mediated through such sources, remained unconfirmed. This key limitation restrains the transferability of findings, and readers should take caution about the verisimilitude of such representation.

This study has two major strengths. First, it analyzed a multi-sourced PC-related information as delivered by the conventional media instead of relying on the accounts of the audience. Second, watching and listening to a variety of men’s statements in the recorded programs was a methodological strength. Indeed, it allowed us to grasp the expression of men’s understanding of existing medical information as related to their experiential knowledge about PC. It was particularly relevant for the audience located in African Francophone countries, thus corroborating our statements about the global impact of French media as a source requiring particular attention from cancer educators and policy makers.

## Conclusion

Since we have virtually no data on how these various media messages on PC are being received, the effect of French media on PC awareness and literacy is unknown and must be studied as the stakes are high. We would argue that health professionals should advocate for improved health literacy regarding PC^68^ and encourage the media’s attention to the inherent diversity of the at-risk male population. Since ethnic background imprints cultural views of PC and men’s sense of masculinity and manhood,^76^ it is important to appreciate ethnic and cultural diversity when designing public education on PC in any country. Media production of mass education material should be more socially and culturally inclusive and gender sensitive to impact the buy-in by men.

## Ethical approval

Not applicable. No human subjects participated in this study

## Competing interests

The authors declare that there is no conflict of interests.

## Disclaimer

The authors claim that no part of this manuscript has been copied from other sources.

## Funding

Union for International Cancer Control-Yamagiwa-Yoshida Memorial International Study Grant-Japan National Committee for UICC and Kyowa Hakko Kogyo Co. Ltd., Japan, earned by the first author. Financial support was also provided by Ryerson University, Research Assistant Program scholarship earned by the second author and the Faculty of Community Services, Writing Week Initiative, granted to the first author for publication support.

## Authors’ contributions

The first and eighth authors contributed to the design of the work. The first author conducted the data retrieval. The first, second, third, fourth, fifth and sixth authors contributed to the interpretation of data for the work; and drafting the manuscript. The seventh author and other authors contributed to the analysis and interpretation of data for the manuscript, as well as approved the final version to publish.

## Acknowledgments

The authors gratefully acknowledge Dr. Corinne Hart, Dr. Manon Lemonde and Dr. Christian Bergeron for reviewing an early draft of this paper, as well as Dr. Sylvia Novac for editing it. We also thank Shahilaa Devaraja for her help in the literature review. Our special Merci beaucoup to the librarians at the Mediathèque, François Mitterrand site-French National Library for their inestimable support during the material retrieval work.

## References

[1] Jensen JD, Moriarty CM, Hurley RJ, Stryker JE (2010). Making sense of cancer news coverage trends: a comparison of three comprehensive content analyses. J Health Commun.

[2] Slater MD, Long M, Bettinghaus EP, Reineke JB (2008). News coverage of cancer in the United States: a national sample of newspapers, television, and magazines. J Health Commun.

[3] Guenther L, Froehlich K, Milde J, Heidecke G, Ruhrmann G (2015). Effects of Valenced media frames of cancer diagnoses and therapies: quantifying the transformation and establishing of evaluative schemas. Health Commun.

[4] Kivits J, Hanique M, Jacques B, Renaud L (2014). L’appropriation de l’information mediatique au sujet de la prevention et du depistage des cancers. Le Temps des medias.

[5] Barber KR, Shaw R, Folts M, Taylor DK, Ryan A, Hughes M (1998). Differences between African American and Caucasian men participating in a community-based prostate cancer screening program. J Community Health.

[6] Clarke JN (1999). Prostate cancer’s hegemonic masculinity in select print mass media depictions (1974-1995). Health Commun.

[7] American Society of Clinical Oncology (ASCO). Prostate Cancer: Screening, 2017. Available from: https://www.cancer.net/cancer-types/prostate-cancer/screening. Accessed December 27, 2017.

[8] Olivier C, Bernard M. Biomarkers of aggressiveness in prostate cancer. In: Spiess PE, ed. Prostate Cancer - Diagnostic and Therapeutic Advances. Rijeka, Croatia: InTech; 2011. p. 3-20.

[9] Jouin A, Mirabel X, Rault E, Reich M, Lartigau E (2015). es nouvelles technologies en radiotherapie : RCMI, stereotaxie, hadrontherapie… quel interet medical et quelles consequences psychologiques pour les patients ?. Psycho-Oncologie.

[10] American Society of Clinical Oncology (ASCO). Prostate Cancer: Treatment, 2017. Available from: https://www.cancer.net/cancer-types/prostate-cancer/treatment-options. Accessed December 27, 2017.

[11] Jung M (2014). Determinants of health information-seeking behavior: implications for post-treatment cancer patients. Asian Pac J Cancer Prev.

[12] Cutilli CC (2010). Seeking health information: what sources do your patients use?. Orthop Nurs.

[13] Ferrante JM, Shaw EK, Scott JG (2011). Factors influencing men’s decisions regarding prostate cancer screening: a qualitative study. J Community Health.

[14] Gibson L, Tan AS, Freres D, Lewis N, Martinez L, Hornik RC (2016). Nonmedical information seeking amid conflicting health information: negative and positive effects on prostate cancer screening. Health Commun.

[15] Miele R, Clarke J (2014). “We remain very much the second sex”: the constructions of prostate cancer in popular news magazines, 2000-2010. Am J Mens Health.

[16] Halpin M, Phillips M, Oliffe JL (2009). Prostate cancer stories in the Canadian print media: representations of illness, disease and masculinities. Sociol Health Illn.

[17] Zanchetta MS, Byam AA, Solomon D, Jalili K, Haag C, Tallarico S (2017). Reports on boys’, youth’s and men’s health in Canadian newspapers: Now what?. Health Promot Perspect.

[18] Hornik R, Parvanta S, Mello S, Freres D, Kelly B, Schwartz JS (2013). Effects of scanning (routine health information exposure) on cancer screening and prevention behaviors in the general population. J Health Commun.

[19] Hong SJ, You KH (2016). The effects of experienced uncertainty and patients’ assessments of cancer-related information-seeking experiences on fatalistic beliefs and trust in physicians. Health Commun.

[20] Bravo CA, Hoffman-Goetz L (2017). Social media and men’s health: a content analysis of Twitter conversations during the 2013 Movember campaigns in the United States, Canada, and the United Kingdom. Am J Mens Health.

[21] Basch CH, Menafro A, Mongiovi J, Hillyer GC, Basch CE (2017). A content analysis of YouTube™ videos related to prostate cancer. Am J Mens Health.

[22] White A (2011). The state of men’s health in Europe - extended report.

[23] Vongmany N, Bousquet PJ. Les cancers en France - Edition 2015. France: Institut national du Cancer; 2015. Available from: http://www.e-cancer.fr/Expertises-et-publications/Catalogue-des-publications/Les-cancers-en-France-Edition-2015. Accessed May 16, 2016.

[24] Xu S, Markson C, Costello KL, Xing CY, Demissie K, Llanos AA (2016). Leveraging social media to promote public health knowledge: example of cancer awareness via Twitter. JMIR Public Health Surveill.

[25] Chevrel S (2016). Cancer et hasardUne derive mediatique passee au crible. Les Tribunes de la sante.

[26] Culture RP. Peut-on encore croire a l’information medicale et scientifique? 2017. Available from: http://culture-rp.com/2017/12/06/peut-on-encore-croire-a-linformation-medicale-et-scientifique/. Accessed December 28, 2017.

[27] World Health Organization. National cancer control programs. Available from: http://www.who.int/cancer/nccp/en/. Accessed May 18, 2013.

[28] Leray C. L’analyse de contenu: De la Theorie a la Pratique: La Methode Morin-Chartier [The content analysis: From theory to practice: The Morin-Chartier’s Method]. Quebec, Qc: Presses de l’Universite du Quebec; 2008.

[29] Renaud L. Les medias et le façonnement des normes en matiere de sante [Media and the construction of health norms]. Quebec, Qc: Presses Universitaires du Quebec; 2007.

[30] Flament C. Aspects peripheriques des representations sociales. In: Guimelli C, ed. Structures et transformations des representations [Structures and transformations of representations]. Lausanne: Delachaux & Niestle; 1994. p. 139-41.

[31] Abric JA (2001). Pratiques sociales et representations [Social practices and representations].

[32] Jodelet D. Representations sociales: un domaine en expansion. In: Jodelet D, ed. Les representations sociales [The social representations]. 6th ed. Paris: Presses Universitaires de France; 1999. p. 47-68.

[33] C’est au programme [television broadcast]. France: France 2; 2011.

[34] Sante: le flair contre le cancer [television broadcast]. France: France 3; 2013.

[35] Depistage du cancer de la prostate: plateau d’analyse d’Elisabeth de Pourquery [television broadcast]. France: France 3; 2012.

[36] Le magazine de la sante [television broadcast]. France: France 5; 2011.

[37] Le depistage systematique de la prostate fait polemique [television broadcast]. France: France 3; 2012.

[38] Le cancer de la prostate [radio podcast]. France: Radio France Internationale; 2012.

[39] La journée nationale de la prostate. Le cancer de la prostate [radio podcast]. France: Kantar Media; 2012.

[40] À la veille de la 7eme journee nationale de la prostate [radio podcast]. France: Radio France Internationale; 2011.

[41] Questions sur les nouvelles armes anti-cancer, les therapies ciblees et le melanome, les cancers du sein et de la prostate [radio podcast]. France: Radio France Internationale; 2011.

[42] Peyromaure M. Depistage du cancer de la prostate: les autorites refusent le progres. Le Figaro. May 16, 2012. Available from:http://www.lefigaro.fr/mon-figaro/2012/05/16/10001-20120516ARTFIG00595-cancer-de-la-prostate-les-autorites-refusent-le-progres.php. Accessed May 5, 2013.

[43] Benkimoun P. Le depistage du cancer de la prostate par test sanguin serait inutile voire nocif. Le Monde. April 4, 2012. Available from: http://www.lemonde.fr/planete/article/2012/04/04/le-depistage-du-cancer-de-la-prostate-par-test-sanguin-serait-inutile-voire-nocif_1680214_3244.html. Accessed May 4, 2013.

[44] Prigent A. Cancer de la prostate: identifier les formes agressives. Le Figaro. May 10, 2012. Available from: http://sante.lefigaro.fr/actualite/2012/05/10/18169-cancer-prostate-depistage-cible. Accessed May 6, 2013.

[45] Mascret D. L’exces de vitamines peut etre dangereux. Le Figaro. October 31, 2011. Available from: http://sante.lefigaro.fr/actualite/2011/10/31/15252-lexces-vitamines-peut-etre-dangereux. Accessed May 4, 2013.

[46] Freour P. L’IRM pour detecter les “vrais” cancers de la prostate. Le Figaro. December 11, 2012. Available from: http://sante.lefigaro.fr/actualite/2012/12/10/19543-lirm-pour-detecter-vrais-cancers-prostate. Accessed May 6, 2013.

[47] Cabut S. Cancer de la prostate: un nouveau traitement prometteur. Le Figaro. July 1, 2011. Available from: http://www.lefigaro.fr/sciences/2011/07/01/01008-20110701ARTFIG00648-cancer-de-la-prostate-un-traitement-prometteur.php. Accessed May 15, 2013.

[48] Une moustache contre le cancer de la prostate. Le Parisien. November 5, 2012. Available from: http://www.leparisien.fr/espace-premium/air-du-temps/une-moustache-contre-le-cancer-de-la-prostate-05-11-2012-2292589.php. Accessed May 15, 2013.

[49] Houdart P, Malye F, Vincent J. Cancer de la prostate: la polemique. Le Point. July 8, 2011. Available from: http://www.lepoint.fr/sante/cancer-de-la-prostate-la-polemique-29-11-2007-1350755_40.php. Accessed May 15, 2013.

[50] L’interet d’un depistage systematique du cancer de la prostate n’est pas démontré. Le Point. April 3, 2012. Available from: http://www.lepoint.fr/sante/l-interet-d-un-depistage-systematique-du-cancer-de-la-prostate-n-est-pas-demontre-03-04-2012-1448057_40.php. Accessed May 15, 2013.

[51] Braly JP. Cancer de la prostate: faut-il toujours operer? La Recherche. October 1, 2012. Available from: http://www.larecherche.fr/actualite/sante/cancer-prostate-faut-il-toujours-operer-01-10-2012-91681. Accessed May 15, 2013.

[52] Jeanblanc A. Prostate: oubliez la mesure du PSA en l’absence de symptomes. Le Point. March 13, 2012. Available from: http://www.lepoint.fr/editos-du-point/anne-jeanblanc/prostate-oubliez-la-mesure-du-psa-en-l-absence-de-symptomes-13-03-2012-1440868_57.php. Accessed May 15, 2013.

[53] Jeanblanc A. Le PSA n’est plus le juge de paix du depistage du cancer de la prostate. Le Point. April 4, 2012. Available from: http://www.lepoint.fr/editos-du-point/anne-jeanblanc/depistage-du-cancer-de-la-prostate-inutile-de-doser-systematiquement-le-psa-04-04-2012-1448181_57.php. Accessed May 15, 2013.

[54] Desgrandchamps F, Hennequin C (2013). Faut-il traiter les cancers de la prostate?. Rev Prat.

[55] Sargueil S. XMRV: un virus accusé de tous les maux. La Recherche. April 1, 2013. Available from: http://www.larecherche.fr/savoirs/sante/xmrv-virus-accuse-tous-maux-01-04-2013-99422. Accessed May 15, 2013.

[56] Cancer et circoncision. La Recherche. 2012 May 1. Available from: http://www.larecherche.fr/actualite/sante/cancer-circoncision-01-05-2012-91157. Accessed May 15, 2013.

[57] Les vertus des vitamines et des oxydants remises en cause. Le Point. October 17, 2011. Available from: http://www.lepoint.fr/sante/les-vertus-des-vitamines-et-des-oxydants-remises-en-cause-17-10-2011-1385409_40.php. Accessed May 15, 2013.

[58] Espoir contre le cancer de la prostate. Le Parisien. 2012 May 21; Sect. Le rendez-vous sante, p. 33 (col. 1).

[59] Des ultrasons contre le cancer de la prostate. Le Parisien. 2012 Apr 23; Sect. Le rendez-vous santé, p. 42 (col. 5).

[60] Prudence avec les compléments alimentaires. Le Parisien. Februar 28, 2013. Available from: http://www.leparisien.fr/espace-premium/actu/prudence-avec-les-complements-alimentaires-28-02-2013-2604755.php. Accessed May 13, 2013.

[61] Rigollet C. Chlordecone, ce poison autorise. Le Point. December 1, 2011. Available from: http://www.lepoint.fr/villes/chlordecone-ce-poison-autorise-15-12-2011-1408561_27.php. Accessed May 13, 2013.

[62] Massonnet C. Un test rend la radiotherapie plus sûre. Le Parisien. 2012 Nov 7; Sect. Actualité, p. 9. Available from: http://www.leparisien.fr/espace-premium/air-du-temps/un-test-rend-la-radiotherapie-plus-sure-07-11-2012-2299177.php. Accessed May 20, 2013.

[63] Soloway MS (2014). Re: overdiagnosis and overtreatment in cancer: an opportunity for improvement. Eur Urol.

[64] Fitzpatrick JM, Kirby RS, Brough CL, Saggerson AL (2009). Awareness of prostate cancer among patients and the general public: results of an international survey. Prostate Cancer Prostatic Dis.

[65] Zanchetta M, Monteiro M, Kaszap M, Gorospe IVF, Pilon R. Renewing perspectives on men’s literacy on prostate cancer and engagement along the disease continuum. In: Spiess PE. Prostate cancer - diagnostic and therapeutic advances. Rijeka, Croatia: InTech; 2011. p. 37-80.

[66] Halverson JL, Martinez-Donate AP, Palta M, Leal T, Lubner S, Walsh MC (2015). Health Literacy and Health-Related Quality of Life Among a Population-Based Sample of Cancer Patients. J Health Commun.

[67] Koo K, Shee K, Yap RL (2018). News Media Analysis of the United States Preventive Services Task Force and American Urological Association Prostate Cancer Screening Guidelines. Urol Pract.

[68] Zanchetta MS, Monteiro MS, Gorospe FF, Pilon RS, Pena A (2010). Ideas of masculinities in Latin America countries and their influences on immigrant men’s attitudes toward health, prostate cancer prevention: an analysis of literature. J Mens Health.

[69] Gigerenzer G, Mata J, Frank R (2009). Public knowledge of benefits of breast and prostate cancer screening in Europe. J Natl Cancer Inst.

[70] Miyawaki R, Shibata A, Ishii K, Oka K (2017). News Coverage of Cancer in Japanese Newspapers: A Content Analysis. Health Commun.

[71] Rains SA, Tukachinsky R (2015). Information Seeking in Uncertainty Management Theory: Exposure to Information About Medical Uncertainty and Information-Processing Orientation as Predictors of Uncertainty Management Success. J Health Commun.

[72] Martinez-Perez B, de la Torre-Diez I, Lopez-Coronado M (2013). Mobile health applications for the most prevalent conditions by the World Health Organization: review and analysis. J Med Internet Res.

[73] Bhatt JR, Klotz L (2016). Overtreatment in cancer - is it a problem?. Expert Opin Pharmacother.

[74] Zanchetta MS, Cognet M, Lam-Kin-Teng MR, Dumitriu ME, Renaud L, Rheaume J (2016). From early detection to rehabilitation in the community: reading beyond the blog testimonies of survivors’ quality of life and prostate cancer representation. Health Qual Life Outcomes.

[75] Organisation Internationale de la Francophonie. Welcome to the International TV5 Monde. Available from: http://www.tv5monde.com/cms/chaine-francophone/tv5monde/La-chaine/p-5857-Presentation.htm. Accessed May 17, 2013.

[76] Cushman MA, Phillips JL, Wassersug R (2010). The Language of Emasculation: Implications for Cancer Patients. Int J Mens Health.

